# Atomic Hydrogen Interaction with Transition Metal Surfaces: A High-Throughput Computational Study

**DOI:** 10.1021/acs.jpcc.4c06194

**Published:** 2024-11-16

**Authors:** Miquel Allés, Ling Meng, Ismael Beltrán, Ferran Fernández, Francesc Viñes

**Affiliations:** Departament de Ciència de Materials i Química Física and Institut de Química Teòrica i Computacional (IQTCUB), Universitat de Barcelona, c/Martí i Franquès 1-11, Barcelona 08028, Spain

## Abstract

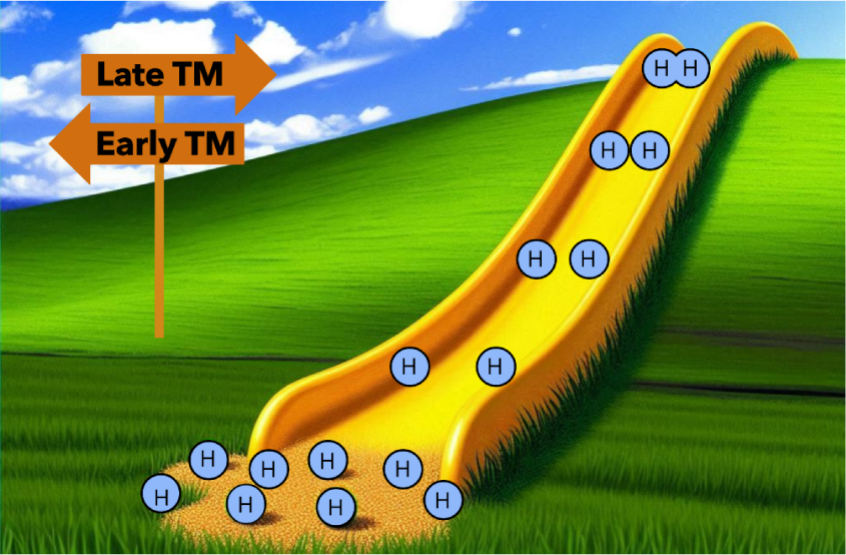

Hydrogen adatoms are involved in many reactions catalyzed by Transition Metal (TM) surfaces, such as the Haber–Bosch process or the reverse water gas shift reaction, key to our modern society. Any rational improvement on such a catalyst requires an atomistic knowledge of the metal↔hydrogen interaction, only attainable from first-principles calculations on suited, realistic models. The present thorough density functional theory study evaluates such H interaction at a low coverage on most stable surfaces of *bcc*, *fcc*, and *hcp* TMs. These are (001), (011), and (111) for *bcc* and *fcc* TMs and (0001), (101̅0), and (112̅0) for *hcp*, covering 27 TMs and 81 different TM surfaces in total. In general terms, the results validate, while expanding, previous assessments, revealing that TM surfaces can be divided into two main groups, one in the majority where H_2_ would be thermodynamically driven to dissociate into H adatoms, located at heights of ∼0.5 or ∼1.0 Å, and another for late TMs, generally with a *d*^10^ electronic configuration, where H_2_ adsorption with no dissociation would be preferred. No trends in H adsorption energies are found down the groups, but yes along the *d* series, with a best linear adjustment found for the *d*-band center descriptor, especially suited for close-packed *fcc* and *hcp* TMs surfaces, with a mean absolute error of 0.15 eV. Gibbs free adsorption energies reveal a theoretical volcano plot where *fcc* TMs are best suited, but with peak Pt performance displaced due to dispersive force inclusion in the method. Still, the volcano plot with respect to the experimental logarithm of the exchanged current density polycrystalline data is far from being valid for a quantitative assessment, although useful for a qualitative screening and to confirm the trends computationally observed.

## 1 Introduction

Transition metals (TMs) have been essential elements for mankind development since copper (Cu) manufacturing, estimated at *ca.* 5000 BC. A large variety of physical, mechanical, and chemical properties of TMs have paved the way for their applicability in multiple, diverse fields; ranging from construction,^1^ through healthcare,^2^ up to heterogeneous catalysis.^3^ As far as the latter topic is concerned, TM catalytic properties have been extensively studied for a myriad of reactions of industrial interest, such as the Haber–Bosch reaction for ammonia synthesis,^4^ the Fischer–Tropsch process to chemically synthesize hydrocarbons from synthesis gas or *syngas*,^5^ or the hydrogenation of unsaturated fats for the food industry,^6^ to name a few. In such processes, active TM phases may imply the use of a pure transition metal, *e.g.*, Ru or Ni,^7,8^ although TM alloys are often sought as a way of tuning the catalyst performance and/or resistance, *e.g.*, as found in CoNi or NiFe bimetallics for hydrogenation of hexane to benzene or CO to methane, respectively.^9,10^

Since such elements are often late TMs, and therefore scarce and expensive, their economically viable use is usually driven by maximizing their exposure by nanostructuring, *e.g.*, employing mono- or bimetallic TM-supported clusters or nanoparticles,^11−13^ up to the extreme point of having supported single-atom catalysts.^14−16^ TMs have been used in diverse reactions involving hydrogen (H_2_) apart from the above-commented ones, *e.g.*, using Pt for hydrogenation reactions,^17^ Cu for methanol synthesis,^18^ and Pt, Pd, Ru, or Au for the Reverse Water–Gas Shift (RWGS) reaction,^19^ to name a few more examples. More recently, with the advent of electrocatalysis, TMs such as Pt, Au, and Pd have been extendedly studied, *e.g.*, in the Hydrogen Evolution Reaction (HER),^20−22^ reducing protons to gain H_2_ gas, eventually usable as fuel. Actually, the use of electric power coming from renewable sources allows tagging the product as *green* H_2_, a worldwide pursued target.

Any rational attempt to optimize such TM catalytic systems is, however, handicapped by the need to understand their very chemical interaction with the reactants, intermediates, and products at the atomic level. This knowledge is difficult to be experimentally grasped given the limitations of many experimental techniques, which very frequently deliver only mean field, average information.^23,24^ Here is where computational chemistry can supply such detailed information, combining, at the same time, suited atomistic models and accurate first-principles calculations. Here, Density Functional Theory (DFT) has excelled in the last decades as the weapon of choice when addressing the interaction of atoms and molecules on TM systems, with abundant examples on the accuracy of such a methodology spanning over many different materials.^25−27^ As far as TM surfaces are concerned, it is worth pointing out recent high-throughput studies, aimed at assessing, *e.g.*, the interaction with C atoms, in the context of carbide formation, site blocking, poisoning, or even activity enhancement,^24,28^ or the CO probe molecule interaction with TM surfaces, are key in Surface Science exploration and a main role-player in many reactions of technological relevance.^25^

Another type of interaction that is fundamental in catalysis is that of TM surfaces with H adatoms, playing a key role in any hydrogenation reaction, either photo-, electro-, or thermo-catalyzed. Here, it is worth pointing out that few previous computational studies already addressed this interaction obtaining H adsorption and absorption energies, *e.g.*, in (001) and (011) surfaces of Fe, Mo, Ta, V, and W body-centered cubic (*bcc*) structures, (001) and (111) surfaces of Ag, Au, Cu, Ir, Ni, Pd, Pt, and Rh face-centered cubic (*fcc*) structures, or the (0001) surface on Co, Os, Re, and Ru hexagonal close-packed (*hcp*) structures.^29−31^ Further studies also reported H adsorption energies on the (111) surface of Cr, Fe, Nb, and W, on the (011) surface of Ir, Ni, Pd, and Rh,^32,33^ and on the (011) surface of Ag, Au, Cu, Ni, Pd, and Pt,^34^ yet such studies still missed the (111) surface for the *bcc* structure, (011) surfaces for Ir and Rh structures, and (101̅0) and (112̅0) surfaces for Cd, Hf, Sc, Tc, Ti, Y, Zn, and Zr on *hcp*. On top of that, one must regard that such studies were carried out at different computational levels, which leads to different data sets which are neither uniform nor complete, since they are missing sites, metals, and surfaces, which are potentially key to acquire a complete overview based on which trends can be captured with no uncertainties.

This complete overview is aimed at studying 81 TM surfaces from all TMs having *bcc*, *fcc*, or *hcp* crystal structures,^35,36^ including most stable surfaces with a maximum Miller index of one. The study, carried out at a low H adatom coverage regime, focuses thus on the inherent interaction of H adatoms with the TM surfaces, without being affected by lateral interactions, and thus permits assessing most stable surface or subsurface sites for each metal, the influence of physicochemical descriptors in the H binding such as the *d*-band center,^37^ trends along series and/or groups, the HER energetic costs over the ideal electrode performance, and the assessment of NP performance as a result of averaged surface contributions.

## 2 Computational Details

**Figure 1:**
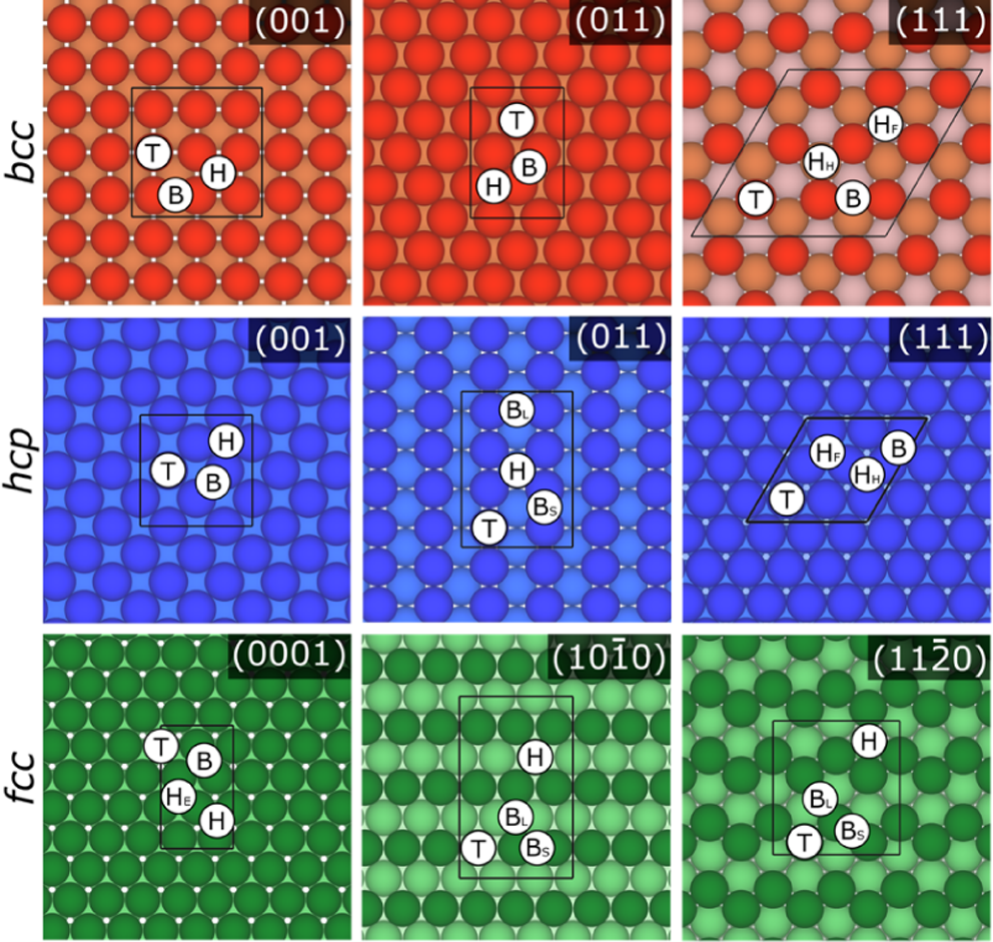
Top views of initial sites for H atoms on the *bcc*, *fcc*, and *hcp* surfaces, separated by rows in red, blue, and green, respectively. The (001), (011), and (111) surfaces are shown for *bcc* and *fcc* structures, while (0001), (101̅0), and (112̅0) surfaces are depicted for *hcp* structures. Top layers are shown as darker colors, while subsurface layers have gradually lighter shades. The black lines define the employed supercells.

As mentioned previously, the present study aims at the high-throughput, systematic investigation of the H interaction with 27 TMs having *fcc* (Ag, Au, Cu, Ir, Ni, Pd, Pt, and Rh), *bcc* (Cr, Fe, Mo, Nb, Ta, V, and W), or *hcp* (Cd, Co, Hf, Os, Re, Ru, Sc, Tc, Ti, Y, Zn, and Zr) crystal structures, thus avoiding Hg, being liquid in normal conditions, Mn, with a complex *bcc* structure composed of Mn_29_ clusters, and La, having a simple hexagonal structure, following a procedure followed earlier.^38,39^ For each TM, its three most stable surfaces with Miller index order maximum of one have been considered; these are (001), (011), and (111) surfaces for *bcc* and *fcc* TMs and (0001), (101̅0), and (112̅0) surfaces for *hcp* TMs. Such surfaces have been modeled using six-layered slab models with imposed periodic boundary conditions, and adding a 10 Å of vacuum on top of it to avoid any significant interaction between slabs, as successfully employed in the past,^25,37,38^ guaranteeing thus energy values to be converged within chemical accuracy of 1 kcal·mol^–1^—*ca.* 0.04 eV— with respect to vacuum and slab widths (see ref. (25) and literature therein). To avoid lateral interactions, supercells have been employed. In particular, *c*(3×3) supercells have been used for *bcc* (001) and *hcp* ( 101̅0) surfaces, *c*(2×2) supercells for *bcc* (011), *fcc* (001), and *hcp* (0001) and (112̅0) surfaces, *p*(3×3) supercells for *bcc* and *fcc* (111) surfaces, and *c*(2×4) supercells for *fcc* (011) surfaces, see Figure 1. By employing them, a similar and comparable H coverage of one atom over eight or nine surface metal atoms —^1^/_8_ or ^1^/_9_ of a monolayer, ML— is achieved. For reference, the H_2_ molecule in vacuum has been optimized **Γ**-point in a large asymmetric unit cell of 9×10×11 Å^3^ dimensions.

DFT calculations have been carried out using the Vienna *Ab Initio* Simulation Package (VASP),^40^ employing the Perdew–Burke–Ernzerhof (PBE) exchange correlation functional,^41^ one of the most accurate in describing TMs bulk and surface geometries and properties.^37,38^ Grimme’s D3 correction was added to include dispersive forces,^42^ proven to be an accurate approach based on previous studies.^25,43^ Projector Augmented Wave (PAW) pseudopotentials have been used to describe the core electrons,^44^ while valence electron densities have been expanded over a plane-wave basis set with kinetic energies up to 415 eV. A Gaussian smearing of 0.2 eV width has been used in the optimizations to accelerate electronic convergence, although final total energies have been gained and extrapolated to zero smearing. Electronic and atomic convergence criteria of 10^–6^ and 10^–5^ eV were employed. The Brillouin zone has been explored on optimal Monkhorst–Pack grids of **k**-points of 3×3×1 dimensions for the employed supercells,^45^ as utilized in the past.^24,25^ Calculations were carried out to be spin-polarized for magnetic Fe, Co, and Ni surfaces. The employed basis set and **k**-point density also provide interaction energies converged up to chemical accuracy, *vide supra*.

For each TM and surface, a hydrogen atom has been allocated over high-symmetry sites of the cell, see Figure 1. These include top (T), bridge (B), and hollow (H) site positions. Notice that, depending on the studied surface, three- or 4-fold hollow positions exist. Aside, three types of 3-fold hollows are differentiated, depending on whether there is a metal atom directly underneath on the first subsurface layer, named H *hcp* (H_H_), in the second subsurface layer, named H *fcc* (H_F_), or with no metal atom underneath, named H empty (H_E_). As far as B sites are concerned, sometimes one has to differentiate between short (B_S_) and large (B_L_) bridge sites. To optimize the H atom position on these adsorption sites, it was consistently initially placed 2 Å above the site. For subsurface sites, the initial position for H is at middle height between surface and first subsurface layer. In all optimizations, all atoms were allowed to fully relax. Once a H ad/absorption minimum was located, the H atom was removed and the reconstructed surface reoptimized, to check whether the surface evolved back to the original structure, or whether other surface minima were found.

The allegedly most stable minima obtained were characterized as such by vibrational frequency analysis, carried out only on the H atom, with frequencies acquired by finite displacements of 0.03 Å in length to construct the corresponding Hessian matrix block, which is later diagonalized to get vibrational frequencies as eigenvalues. By this, all such situations were confirmed to be minima of the potential energy surface. For such minima, the height *h* of the H atom was measured with respect to the mean height of the atomic positions of the surface metal atoms. The obtained frequencies were used to correct the energies by the Zero Point Energy (ZPE), defined as;
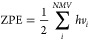
1where *h* is Planck’s constant and *v*_*i*_ is the vibrational frequencies for each Normal Mode of Vibration (NMV). In the case of H_2_ in a vacuum, only the stretching frequency is regarded, while for ad/absorbed H, three NMVs were used, belonging to surface-bonding frustrated translations.

The interaction strength of adsorbed H atoms is here assesed by the adsorption energy *E*_ads_, defined as;
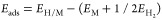
2where *E*_H/M_ is the total energy of the TM surface with the adsorbed H, *E*_M_ is the total energy of the clean TM surface, and  is the total energy of the hydrogen molecule in vacuum. This way, *E*_ads_ is defined with respect to half the energy of the hydrogen molecule, so accounting for the H_2_ bond dissociation, meaning that positive *E*_ads_ values correspond to situations where gas-phase H_2_ would be more stable, while negative *E*_ads_ values imply that it would be energetically more stable to have H adatoms, *i.e.*, that H_2_ would be thermodynamically driven to dissociate on the explored surface. In the case of absorption energies *E*_abs_, the same equation and reference is used.

In order to assess HER capabilities, the Gibbs free energy of H adsorption, Δ*G*_ads_ has been calculated, defined as;
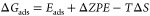
3where Δ*ZPE* is the difference between the ZPE of the adsorbed H and half the ZPE of the H_2_ molecule in vacuum. Finally, *T* is the working temperature, set as a standard temperature of 298.15 K, while Δ*S* is defined as;
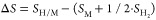
4where *S*_H/M_, *S*_M_, and  are the entropies for hydrogen adsorbed on the metal surface, that of the pristine surface, and of the hydrogen molecule in vacuum, respectively. For , the tabulated value of 130.68 J·mol^–1^·K^–1^ was used, obtained from the National Institute of Standards and Technology (NIST) webbook,^46^ although the present computational estimate of  is 144.68 J·mol^–1^·K^–1^ by using *ab initio* estimates of the rotational, translational, and vibrational partition functions.^47^ Thus, the use of tabulated data brings a minimal impact of 0.06 eV weaker Δ*G*_ads_ at a standard temperature *T* of 298.15 K. The *S*_H/M_ has been obtained from the vibrational entropy *S*_vib_ of the ground state where H is ad/absorbed, taking into account the frustration of translation and rotation modes due to the bonding with the TM surface and so regarding three vibrational modes per H adatom. Thus, *S*_vib_ —without taking into account ZPE, to avoid double-counting— can be estimated as;
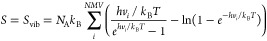
5where *N*_A_ is Avogadro’s number and k_B_ is Boltzmann’s constant. In the case of *S*_M_, it has been neglected, assuming that phonon vibrational entropy for the pristine TM surface and that of the surface having H adatoms are equal. Note that this way of estimating vibrational entropies has been extendedly and successfully used in many electrocatalytic studies in the past.^48−50^

## 3 Results and Discussion

**Figure 2:**
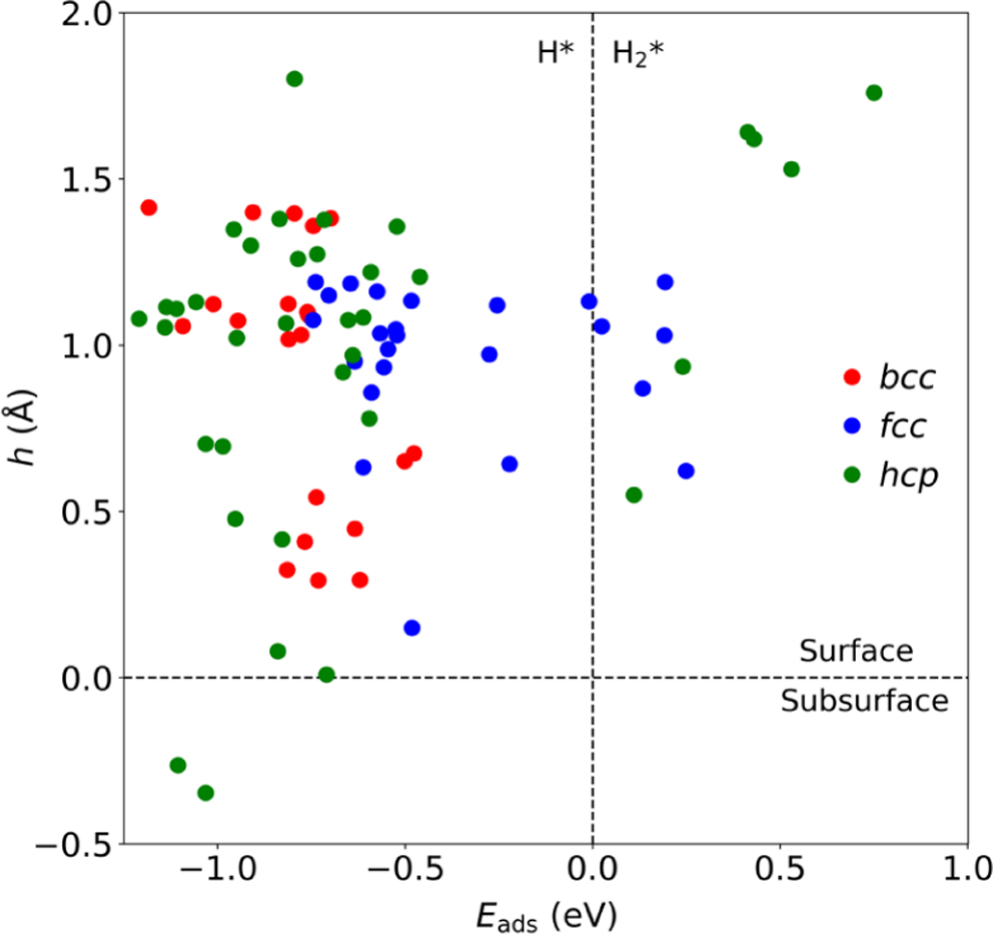
Quadrant plot of H height *h* in Å *vs* the adsorption energy *E*_ads_, in eV, for most stable site as reported in Table 1. Color code as in Figure 1.

After more than 300 DFT optimizations were carried out over the 27 TMs and 81 TM surfaces, the most stable adsorption/absorption energies and heights have been collected in Table 1, while Tables S1–S3 contain the data for all found minima. Note that, at variance with C adatom situations,^24^ H atoms generally prefer to be adsorbed, with only two subsurface cases, these are, Sc and Y subsurface B_L_ sites of (112̅0) surfaces, see Table 1, and *h*/*E*_ads_ quadrant plot in Figure 2. The accentuated surface preference may seem counterintuitive when thinking about the small H size, easily fitting subsurface site voids, but one has to keep in mind that its 1*s*^1^ electronic configuration is not well-adapted for multiple interaction, plus its small size in subsurface voids can be adverse in terms of keeping various simultaneous bond distances with neighboring metal atoms. One should also note that, in some metals like Pd and Ni, subsurface H is found to be stable only when surface H adatom, H*, coverage is high, according to previous DFT studies.^51,52^ Finally, note aside that the listed Δ*G*_ads_ values are larger than *E*_ads_ by between 0.10 and 0.26 eV, after including ZPE and *TS*_vib_, and so, in terms of HER, optimal TM surfaces would be, in theory, those with *E*_ads_ between −0.10 and −0.26 eV, accordingly, although one should consider other factors, see below.

**Table 1:** Most Stable Sites, Adsorption Energies *E*_ads_ and Heights *h* on the Studied TM Surfaces, as well as the Free Energy of Adsorption Δ*G*_ads_

Metal	Surface	Site	*E*_ads_(eV)	Δ*G*_ads_(eV)	h (Å)
Cr	(001)	B	–0.80	–0.57	1.40
Fe	(001)	H	–0.50	–0.38	0.65
Mo	(001)	B	–0.78	–0.56	1.41
Nb	(001)	B	–0.70	–0.48	1.38
Ta	(001)	B	–0.75	–0.51	1.36
V	(001)	H	–0.74	–0.58	0.54
W	(001)	B	–0.91	–0.67	1.40
Cr	(011)	H^a^	–0.81	–0.56	1.13
Fe	(011)	H^a^	–0.81	–0.58	1.02
Mo	(011)	H^a^	–0.76	–0.53	1.09
Nb	(011)	H^a^	–0.95	–0.71	1.07
Ta	(011)	H^a^	–1.01	–0.77	1.12
V	(011)	H^a^	–1.09	–0.85	1.06
W	(011)	H^a^	–0.76	–0.53	1.10
Cr	(111)	B	–0.77	–0.55	0.41
Fe	(111)	B	–0.48	–0.23	0.68
Mo	(111)	B	–0.63	–0.49	0.45
Nb	(111)	B	–0.62	–0.41	0.29
Ta	(111)	B	–0.73	–0.50	0.29
V	(111)	B	–0.82	–0.59	0.33
W	(111)	B	–0.78	–0.55	1.03
Ag	(001)	H	0.25	0.35	0.62
Au	(001)	B	0.02	0.23	1.06
Cu	(001)	H	–0.22	–0.06	0.64
Ir	(001)	B	–0.74	–0.51	1.19
Ni	(001)	H	–0.61	–0.44	0.63
Pd	(001)	B	–0.57	–0.36	1.04
Pt	(001)	B	–0.75	–0.52	1.08
Rh	(001)	B	–0.58	–0.37	1.16
Ag	(011)	B_S_	0.19	0.38	1.19
Au	(011)	B_S_	–0.01	0.20	1.13
Cu	(011)	B_S_	–0.26	–0.04	1.12
Ir	(011)	B_S_	–0.65	–0.43	1.19
Ni	(011)	B_L_	–0.48	–0.28	0.15
Pd	(011)	B_S_	–0.53	–0.32	1.05
Pt	(011)	B_S_	–0.70	–0.48	1.15
Rh	(011)	B_S_	–0.48	–0.28	1.13
Ag	(111)	H_F_	0.19	0.39	1.03
Au	(111)	H_F_	0.13	0.32	0.87
Cu	(111)	H_F_	–0.28	–0.04	0.97
Ir	(111)	H_F_	–0.52	–0.32	1.03
Ni	(111)	H_F_	–0.64	–0.39	0.95
Pd	(111)	H_F_	–0.59	–0.36	0.86
Pt	(111)	H_F_	–0.56	–0.37	0.93
Rh	(111)	H_F_	–0.55	–0.33	0.99
Cd	(0001)	B	0.53	0.70	1.53
Co	(0001)	H_E_	–0.64	–0.39	0.97
Hf	(0001)	H_E_	–1.14	–0.91	1.05
Os	(0001)	H_E_	–0.61	–0.39	1.08
Re	(0001)	H_E_	–0.95	–0.69	1.02
Ru	(0001)	H_E_	–0.65	–0.42	1.08
Sc	(0001)	H_E_	–1.14	–0.91	1.12
Tc	(0001)	H_E_	–0.82	–0.56	1.07
Ti	(0001)	H	–1.21	–0.96	1.08
Y	(0001)	H_E_	–1.06	–0.85	1.13
Zn	(0001)	B	0.43	0.62	1.62
Zr	(0001)	H	–1.11	–0.88	1.11
Cd	(101̅0)	B_S_	0.75	0.92	1.76
Co	(101̅0)	H^b^	–0.60	–0.36	0.78
Hf	(101̅0)	B_S_	–0.96	–0.75	1.35
Os	(101̅0)	B_S_	–0.79	–0.56	1.26
Re	(101̅0)	B_S_	–0.91	–0.67	1.30
Ru	(101̅0)	H^b^	–0.67	–0.45	0.92
Sc	(101̅0)	H^b^	–1.03	–0.80	0.70
Tc	(101̅0)	B_S_	–0.80	–0.57	1.80
Ti	(101̅0)	B_L_	–0.95	–0.71	0.48
Y	(101̅0)	H^b^	–0.99	–0.77	0.70
Zn	(101̅0)	B_S_	0.41	0.62	1.64
Zr	(101̅0)	B_S_	–0.84	–0.65	1.38
Cd	(112̅0)	B_S_	0.24	0.42	0.94
Co	(112̅0)	B_S_	–0.46	–0.25	1.21
Hf	(112̅0)	B_L_	–0.71	–0.48	0.01
Os	(112̅0)	B_S_	–0.74	–0.52	1.27
Re	(112̅0)	B_S_	–0.72	–0.48	1.38
Ru	(112̅0)	B_S_	–0.59	–0.39	1.22
Sc	(112̅0)	B_L_	–1.11	–0.88	–0.26
Tc	(112̅0)	B_S_	–0.52	–0.31	1.36
Ti	(112̅0)	B_L_	–0.84	–0.60	0.08
Y	(112̅0)	B_L_	–1.03	–0.82	–0.35
Zn	(112̅0)	H^c^	0.11	0.31	0.55
Zr	(112̅0)	H^c^	–0.83	–0.59	0.42

a Distorted 3-fold hollow site.

b Distorted bridge-hollow site.

c Distorted bridge-hollow site.

**Figure 3:**
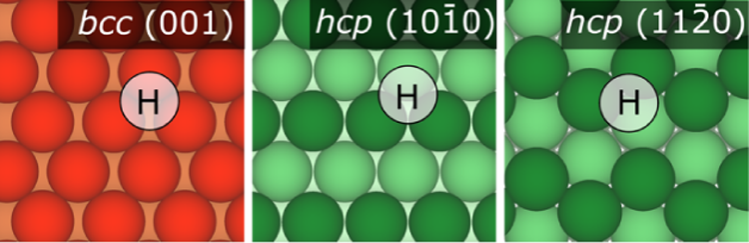
Top view of distorted 3-fold H positions on *bcc* (011), *hcp* (101̅0), and *hcp* (112̅0) surfaces. Color code as in Figure 1.

Analyzing the results by crystallographic types, on *bcc* TMs, B sites are preferred on (001) surfaces except for Fe and V, which prefer H sites, with B *E*_ads_ ranging from −0.70 to −0.91 eV —and heights *h* distant from the surface from 1.36 to 1.41 Å, while H sites display a smaller binding energy spanning from −0.50 (Fe) to −0.74 eV (V) —and *h* values of 0.65 and 0.54 Å, respectively. On (011) surfaces, the most stable site is consistently a distorted H, see Figure 3, where H is 3-fold coordinated, with *E*_ads_ ranging from −0.76 to −1.09 eV and larger heights of 1.02 to 1.13 Å. Finally, for (111) surfaces, the most stable position is B, with *E*_ads_ from −0.48 to −0.82 eV and close height between 0.29 and 0.68 Å, except for W with an *h* of 1.03 Å. On Figure 2 quadrant plot, the above cases are visible with a H* surface preference, with values grouped at two different heights, as already stated.

When analyzing *fcc* TM surfaces, even if they are late TMs compared to *bcc* surfaces, which are normally early TMs, some similarities arise. For instance, the B site is preferred on (001) surfaces as well, with *h* values from 1.04 to 1.16 Å, although with weaker interaction energies, spanning from −0.57 to −0.75 eV, while noble metal Au shows an adsorption energy of 0.02 eV. However, Ag, Cu, and Ni have a preference toward the H site, although with a lower height of *ca.* 0.63 Å, with generally small adsorption energies of 0.25, −0.22, and −0.61 eV, respectively. Note how on Ag the adsorption energy is less preferential, a feature also observed in the literature when comparing C interaction with Au and Ag nanoparticles, implying a, in principle, counterintuitive greater nobility of Ag compared to Au, although understandable due to the deeper *d*-band center of the Ag, and the weak Ag–C coupling, destabilizing the bond and increasing the adsorption energy as already discussed in the literature.^28^ When inspecting (011) surfaces, B_S_ is the most stable site for all TMs, except for Ni, which occupies B_L_. Generally, *h* values range from 1.05 to 1.19 Å, with *E*_ads_ values from 0.19 to −0.70 eV, with Ag again displaying the least favorable interaction. In the case of Ni, the interaction falls within the range, with an *E*_ads_ of −0.48 eV, but H is more distant but with a close height of 0.15 Å. Finally, on the (111) surface, all cases prefer H_F_, with heights between 0.86 and 1.03 Å and moderate *E*_ads_ from 0.19 to −0.64 Å, in accordance with previous studies.^29−33^ All of the above is visually shown in the quadrant plot of Figure 2, with hydrogen liking being on the surface and with two main behaviors either with a preference toward having H* with *E*_ads_ values ranging from −0.5 to −0.7 eV or with small yet positive *E*_ads_ values for more noble *fcc* TMs and surfaces.

**Figure 4:**
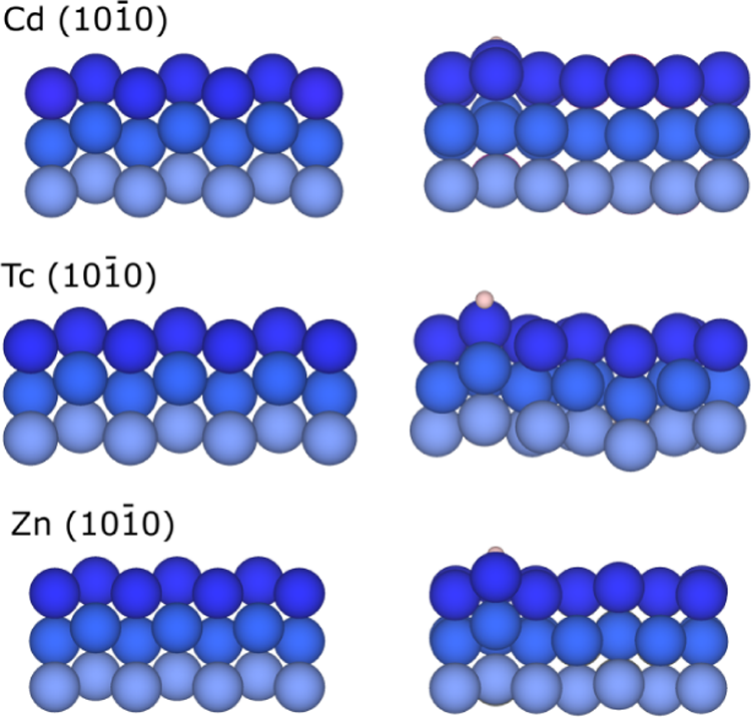
Side view of Cd, Tc, and Zn (101̅0)-distorted surfaces before (left) and after (right) H adsorption. The hydrogen atom is shown as a white sphere, while the rest of color coding is as in Figure 1, yet here each color shade groups two atomic layers; *i.e.*, dark blue represents surface and first subsurface layers.

When inspecting *hcp* TMs, the close-packed (0001) surface is structurally similar to the *fcc* (111) but with a different layer stacking, see Figure 1. For most of the cases, H_E_ is the most stable site, similar to H_F_ sites on *fcc* (111) cases, *i.e.*, with H looking for a surface hollow pocket with minimal subsurface steric repulsion. There, *h* ranges from 0.97 to 1.13 Å with *E*_ads_ spanning from −0.61 to −1.14 eV. In the case of Ti and Zr, the preferred site is still similar to the H site, with an *E*_ads_ of −1.21 and −1.11 eV and an *h* of 1.08 and 1.11 Å, while Cd and Zn show positive *E*_ads_ values of 0.53 and 0.43 eV on a B position with higher adsorptions at 1.53 and 1.62 Å, respectively. On the (101̅0) surface, B_S_ is preferred for Zr, Hf, Tc, Re, Os, Zn, and Cd with *h* from 1.26 up to 1.80 Å and *E*_ads_ between 0.75 and −0.96 eV, being weakest again for Cd and Zn, see above, and strongest for early TMs. It is worth looking at Cd, Tc, and Zn, whereas noticeable deformation of the surface occurs when the H atom is adsorbed, as shown in Figure 4. In the case of *d*^10^ Cd and Zn, it implies a pulling out of the H-bond surface plus neighboring metal atoms, while on Tc, pulling out is mirrored on other empty sections of the slab.

**Figure 5:**
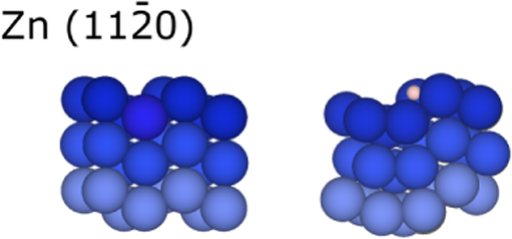
Side view of Zn (112̅0)-distorted surface before (left) and after (right) H adsorption. Color coding as in Figure 4.

Aside from Sc, Y, Ru, and Co, the most stable ones are H-distorted sites, see Figure 3, with *E*_ads_ between −0.60 and −1.03 eV and *h* from 0.70 up to 0.92 Å. The case of Ti is different, with a preference for the B_L_ site at an *h* of 0.48 Å and an *E*_ads_ of −0.95 eV. In the case of the (112̅0) surface, the most stable site is B_S_, with *E*_ads_ values between 0.24 and −0.74 eV and *h* from 0.94 up to 1.38 Å. There are exceptions on early Sc, Y, Ti, and Hf cases, where B_L_ is most stable with *E*_ads_ between −0.71 and −1.11 eV and subsurface heights of −0.26 to −0.35 Å for Sc and Y, while Hf and Ti are essentially in-plane at heights of 0.01 and 0.08 Å, while Zn and Zr prefer H sites with an *E*_ads_ of 0.11 and −0.83 eV and an *h* of 0.55 and 0.42 Å, respectively. Similarly, as for the (101̅0) surface, Figure 5 shows how the Zn (112̅0) surface presents a vertical deformation when the H atom is adsorbed. As per the display on the quadrant plot in Figure 2, the just commented subsurface site preferences are easily spotted, while the rest of the *hcp* situations are concentrated on the surface H* preference quadrant, mostly concentrated at a height of ∼1.00 Å.

The above-described independent results have been compared with some of the most extensive studies available in the literature,^30,31,34^ showing, in any case, a perfect agreement in what concerns the adsorption site preference. Notice that, from the 81 TM surfaces explored here, Ferrin *et al.* studied 30 cases, while by Bai *et al.* and Zhai *et al.*, 20 and six cases were studied, respectively, representing thus, at most, 44.4% of the data set here examined as most of the cases between Ferrin *et al.* and Bai *et al.* are replicated, obtaining similar results. Still, a comparison of *E*_ads_ can be carried out, taking into consideration that, in previous studies, *E*_ads_ was obtained as
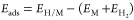
6where the whole H_2_ molecular energy is subtracted, and no ZPE was included. Note as well that Ferrin *et al.* used PW91 functional^53^ on DACAPO^54^ software, defining (2×2) supercells with a higher coverage of ^1^/_4_ ML, a lower cutoff energy of 340 eV for the plane-wave basis set, and generally lighter **k**-point grids of 4×4×1 for the employed supercells. Similarly, Bai *et al.* also used PW91 functional with VASP software, performing adsorption on (3×3) supercells with a coverage of ^1^/_9_ ML, but with a denser **k**-grid of 6×6×1, and a close energy cutoff of 400 eV. Finally, Zhai *et al.* used PBE functional on VASP, replicating (2×2) supercells with a ^1^/_4_ ML, a 5×5×1 **k**-point grid, and a cutoff energy of 450 eV. Still, it is important to notice that none of these previous studies regarded dispersive forces, and Ferrin *et al.* and Bai *et al.* used slabs of four to six layers, with only half allowed to relax, while Zhai *et al.* employed four-layered fully optimized slabs.

**Figure 6:**
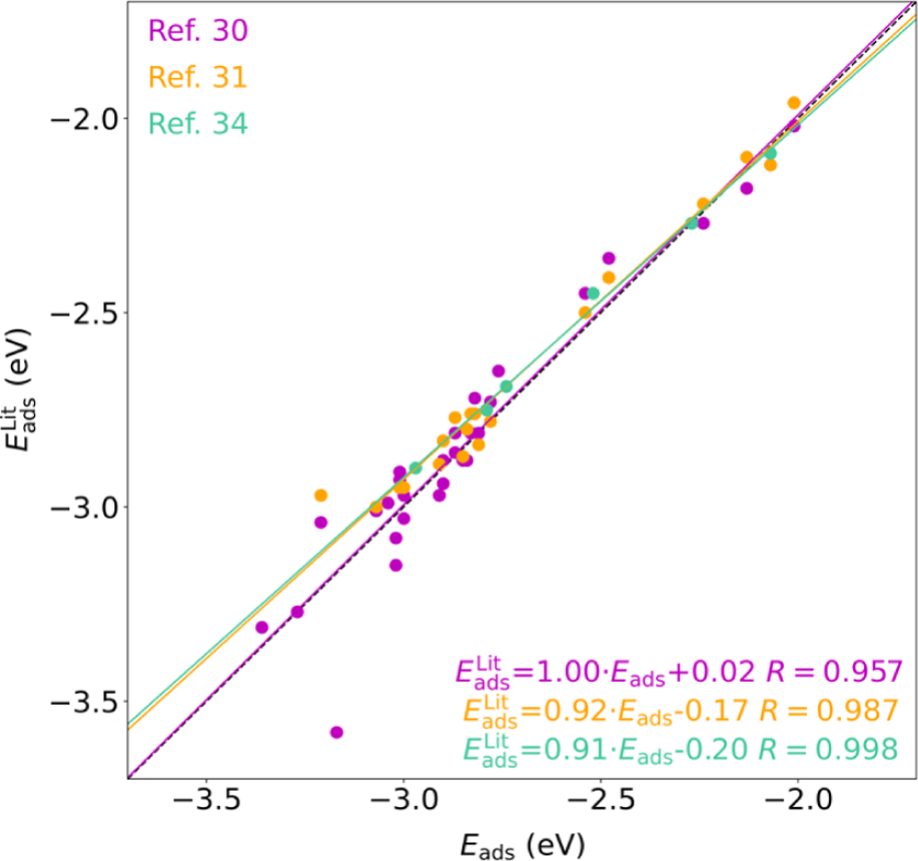
Parity plot comparing presently calculated *E*_ads_ values with those found in the literature (Lit.), including Ferrin *et al.*,^30^ Bai *et al.*,^31^ and Zhai *et al.*(34) The linear correlation equation and regression coefficients are shown color-coded.

With the above in mind, one has to understand that slight variations are to be present when comparing present and past results, as biased by the supercell dimensions (coverage), slab width, number of optimized layers, employed basis set, and **k**-point grids. Still, the parity plot shown in Figure 6 reveals an overall excellent agreement observing divergences, on average, smaller than 0.05 eV, and no study systematically over- or underestimates the bonding strengths. Actually, the linear regressions show very high regression coefficients *R* in all cases above 0.95, slopes very close to unity, and small intercepts below 0.2 eV. If any, the sole most divergent situation is H adsorption on top of the W (001) surface, with a present *E*_ads_ 0.41 eV more stable than the value provided by Ferrin *et al.*,^30^ even if the same bridge adsorption site is found, with *h* values differing by only 0.01 Å. Any attempts to reproduce the previous data have failed as the optimized geometry is essentially the same, and the marked difference is allegedly due to the combined effects on the same direction of a different employed exchange-correlation functional, coverage, **k**-point grid, and plane-wave cutoff. In any case, this particular case seems the exception that confirms the rule that present results are in line with previous studies, further validating them while expanding the present study to more TMs and surfaces.

Having certified the gained minima, the quadrant plot shown in Figure 2 reveals three different behaviors: On the one hand, the largest group displays positive *h* values —between 0.75 and 2.00 Å— implying H adatoms preference, with a negative *E*_ads_ with respect to the H_2_ molecule —ranging from −0.22 to −1.16 eV— unveiling that in such TM surfaces, the H_2_ adsorption and dissociation would be energetically rewarded. Note here that, obviously, adsorbed H_2_, H_2_*, would need to overcome a dissociation energy barrier, although the majority of cases imply *E*_ads_ between −0.5 and −1 eV, see Figure 2, making the reaction step quite energetically downhill, and so expecting an early transition state with low-energy barriers, as found, *e.g.*, in Pd, Pt, and Cu surfaces when studied in the literature,^55,56^ a point that supports a common approach of considering such a step and a barrierless process.

With an *E*_abs_ larger than −1 eV, one has another quadrant, with a preference toward H_2_ dissociation, but having subsurface H atoms, being the two commented Sc and Y (112̅0) surfaces. For such situations to happen, one has to regard that both H_2_ dissociation and H subsurface sinking energy barriers should be small, although the latter is foreseeable, keeping in mind the small size of the H atom. Finally, the third group implies positive *h* and *E*_ads_ values, implying an energetic preference for H_2_. Here, it is worth underscoring Au surfaces, and to a lesser extent, Ag surfaces, as systems where having H* or H_2_* species would be energetically competitive, while H_2_* is clearly preferred for Zn and Cd *d*^10^ TM surfaces. Note that the closest values to *E*_ads_ of zero eV in the first quadrant belong to Cu and that with a *d*^10^*s*^1^ electronic configuration resembles other elements of the same group.

**Figure 7:**
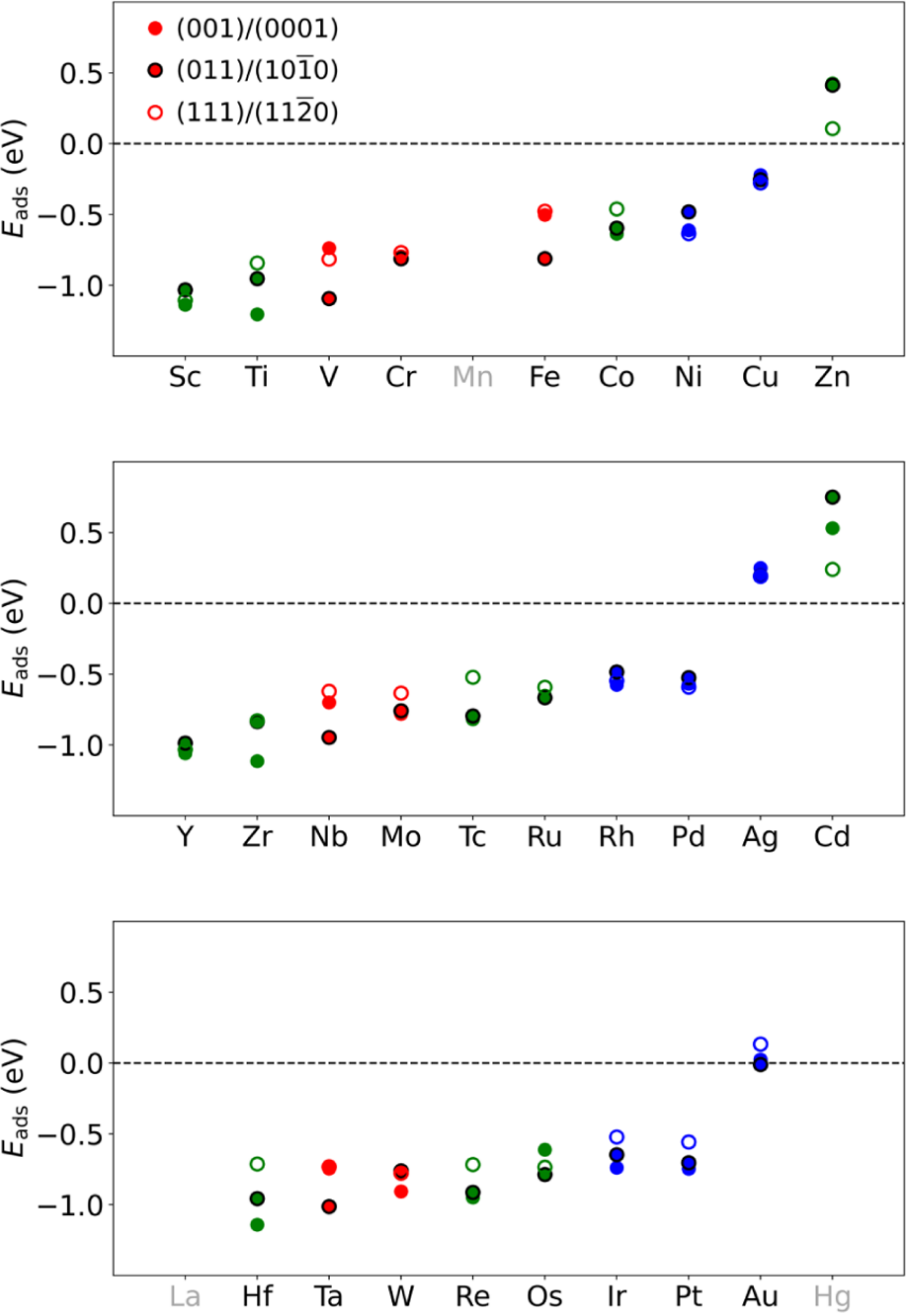
Computed *E*_ads_ evolution on the 81 TM surfaces along the 3*d* (top), 4*d* (middle), and 5*d* (bottom) series. The *bcc*, *fcc*, and *hcp* crystallographic structures are color-coded as in Figure 1, with different filling and outer-rim to differentiate different surface terminations.

Having clearly identified and characterized the most stable sites for H* on the 81 TM surfaces, it is worth trying to capture bonding strength trends along the periodic table *d* series and groups. This is shown in Figure 7, clearly revealing that *E*_ads_ has a clear trend along the 3*d*, 4*d*, or 5*d* series in that it gets less negative, up to positive values for groups XI (Cu, Ag, and Au) and XII (Zn, Cd). However, there is no crystal-clear trend down a given group, nor among the different exposed surfaces for a given TM. Thus, the main descriptor governing the interaction of H on TM surfaces must be a property that continuously changes along the *d* series. In this sense, the present *E*_ads_ was linearly correlated to a series of energetic or electronic structure descriptors proposed in the literature, including the TM surface energy γ,^57^ the work function ϕ,^58^ the *d*-band center ε_d_,^59^ the corrected *d*-band center ,^60^ and the highest Hilbert transform *d*-band peak ε_u_.^61^ Notice that such correlations are mathematically grounded correlating a continuous property with a continuous descriptor, and along this line, one avoids correlating continuous interaction energy values with descriptor discrete values, such as the coordination number or the number of *d* electrons.

**Figure 8:**
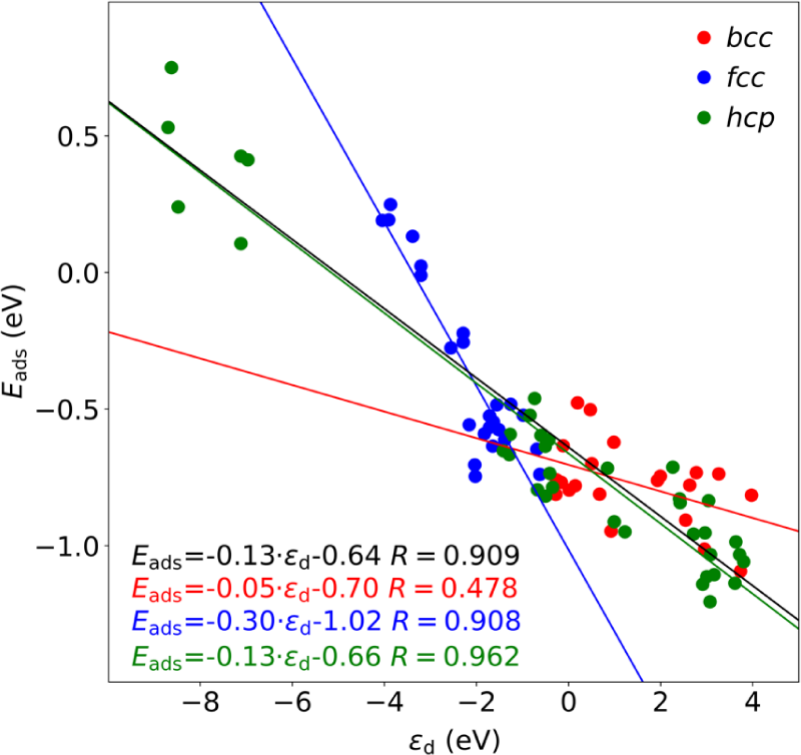
Linear correlation of *E*_ads_*vs* the *d*-band center ε_d_ shown in black. The linear correlations for different crystallographic structure subsets are shown color-coded as in Figure 1.

The descriptor values were collected from previous works, computed at the very same calculation level as the present study.^37,62^ As in previous assessments, the best correlation is gained for the *d*-band center, with an overall regression coefficient of 0.909 and a Mean Absolute Error (MAE) of 0.15 eV, see Figure 8. The rest of correlations, shown in Figures S1–S4, are worse than ε_d_, being actually ϕ the worst-case descriptor, with an *R* of 0.250 and an MAE of 0.31 eV, unveiling that charge transfer to H does not seem to be key in the interaction. Focusing on the best descriptor so far, ε_d_, when it comes to the different crystallographic structures subsets, as already observed for C adatoms,^24^ the adequacy of the *d*-band center is maximal for close-packed situations, such as *hcp* and *fcc* TM surfaces, with *R* values of 0.962 and 0.908, respectively, and MAEs of 0.12 and 0.10 eV, respectively, but it loses adequacy for *bcc* TMs, with a reduced *R* value of 0.478 and a concomitantly increased MAE of 0.11 eV. Still, from all the considered descriptors, the *d*-band center excels due to its precision and simplicity.

**Figure 9:**
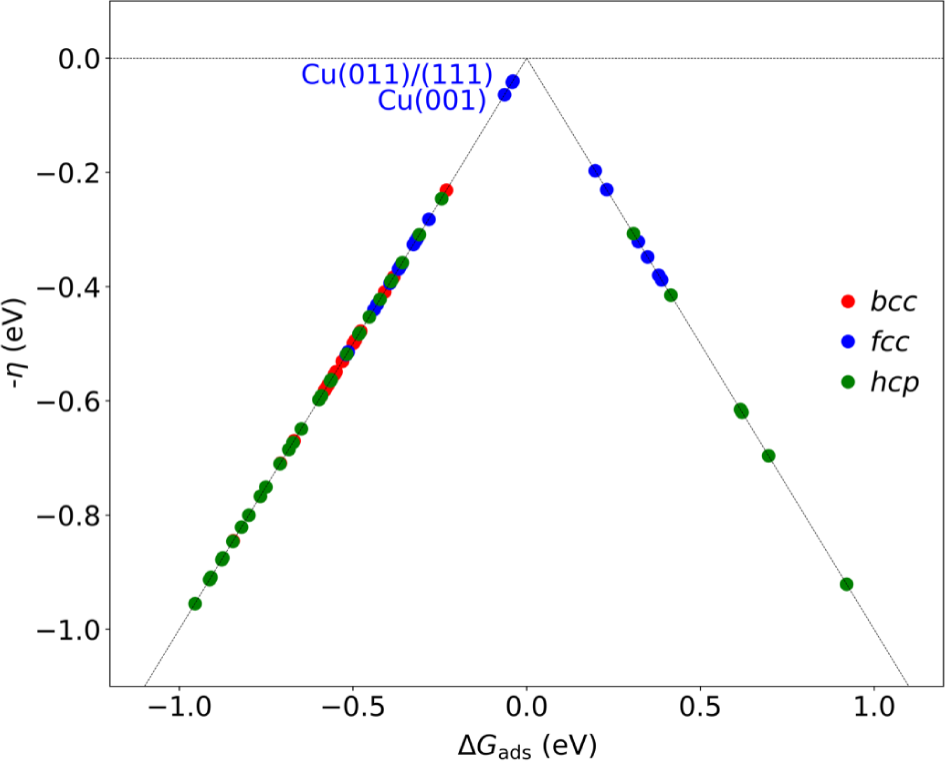
Volcano plot of overpotential *–*η *vs* the computed absolute value of Δ*G*_ads_ for the most stable H* position. Color code as in Figure 1.

Last but not least, one could use the computed data to assess the HER performance of the TM surfaces. This is carried out here using the computed Δ*G*_ads_ values, listed in Table 1, and assuming a Volmer–Heyrovsky mechanism, as usually approached in the literature as a first approach.^48,63,64^ The theoretical overpotentials for the different systems are defined as η = |Δ*G*_ads_|/*e*, and the volcano plot is gained by plotting *–η vs* Δ*G*_ads_. The resulting volcano plot, shown in Figure 9, reveals that *bcc* TM surfaces bind H* too strongly, and so most of *hcp* TM surfaces, while late *hcp* TM surfaces bind too weakly; actually, the *fcc* TMs are consistently closer to the optimal zero overpotential η, as found in experiments.^65−68^ Note that, however, the present calculations would point out Cu surfaces to be optimal for HER, with Δ*G*_ads_ values closer to zero, see Table 1, although is well-known that Pt is best suited,^63,69^ which normally gives Δ*G*_ads_ values on Pt(111) close to zero when carried out at the PBE level.^70^ This is simply explained here by the effect of dispersive forces, known to reduce adsorption energies of simple atoms/molecules by 0.2–0.4 eV.^71^ With this in mind, then Pt, Pd, Rh, Ir, and Re surfaces turn out to be optimal for HER applications, in line with the experimental observation based on the logarithm of the exchanged current density, log(*i*_0_).^67^

However, the same experiments reveal, for a few cases, how for Pt and Au, the (111) surfaces show poorer performance with respect to polycrystalline data, while the opposite happens with Ag. One could benefit from TM Wulff shapes as derived from computed surface energies to average the Δ*G*_ads_ according to the fraction of the exposed surfaces as a more suited approach to polycrystalline data.^61^ However, by doing so, the changes in the volcano plot are minimal, see equivalent volcano plot in Figure S5, a somewhat foreseeable aspect since (111) surfaces are dominant on such *fcc* TMs. Thus, the difference in activity between single-crystal surfaces and polycrystalline samples may well arise from a higher exposure of surface active sites per material mass unit and is influenced as well by other factors playing a role, like mass transport limitation, which is found to be key for HER on Pt.^72^

**Figure 10:**
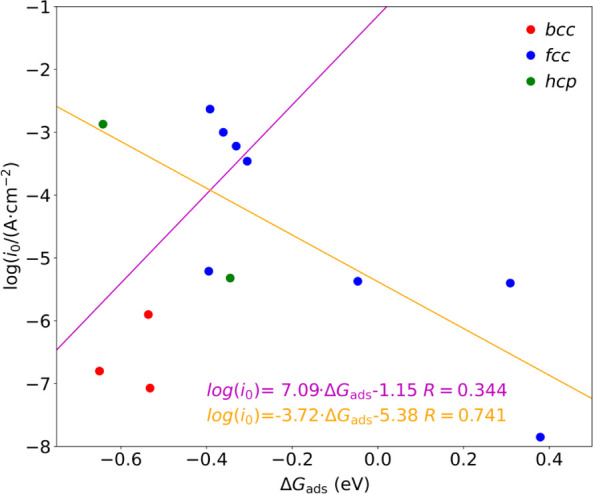
Experimental logarithm of the exchanged current density log(*i*_0_) on polycrystalline data *vs* the PBE-D3 H* Δ*G*_ads_ estimates, Wulff-shaped averaged. Values left and right from the Pt case apex are shown in red and blue, respectively, with both sides linear regressions and *R* values.

The previous corollary underscores that H* Δ*G*_ads_ is not the only factor for HER on TM electrocatalysts, and actually, a proper volcano plot with Wulff-averaged Δ*G*_ads_ estimates does not show a sufficient direct proportionality, see Figure 10. *All that is gold does not glitter*, and the widely used volcano approach to first assess the HER capabilities should take this evaluation as merely qualitative; even when taking Pt as the apex change of the trend, the linear regression trends at smaller and larger Δ*G*_ads_ values feature low *R* values of 0.344 and 0.741 and, concomitantly, large MAE errors on log(*i*_0_) of 1.653 and 0.945, respectively, even if the arrangement of the TM crystallographic types follows that in Figure 9. Thus, such an approach should be taken with caution and as a mere way of discarding certain systems with excessive change in Δ*G*_ads_ from the Pt reference, rather than of a way of quantitatively assessing the HER performance, given the excessive error in predicting the exchanged current density by 1–2 orders of magnitude.

## 4 Conclusions

The present study thoroughly explored the interaction of H atoms on 81 TM surfaces at a low coverage regime, finding that most stable sites are generally on the surface, with the exception of absorption sites on Sc and Y (112̅0) surfaces. The majority of adsorption situations imply a preference toward H_2_ dissociation in most of the other TM surfaces, H being typically placed either at ∼0.5 Å or at ∼1.0 Å. Only late TMs, belonging mostly to *fcc* or *hcp* crystallographic structures with a *d*^10^ electronic configuration, display a preference toward H_2_ molecules. The present description of the adsorptive landscape validates and expands previous literature on the matter, allowing for an evaluation of the interaction of H atoms with TM surfaces along the *d* series and periods, with no consistent trend along the periods, but a clear trend along the *d* series, in that the H interaction energy weakens with the number of *d* electrons. An evaluation of possible descriptors reveals a rather good correlation with the *d*-band center ε_d_, specially suited for close-packed TM surfaces with either *fcc* or *hcp* crystallographic structures, with an excellent overall MAE of solely 0.15 eV.

The computed Gibbs free energies of H adsorption Δ*G*_ads_ gained at a pH of 0 and a potential *U* of 0 V are used to acquire the volcano plot trend by assuming a Volmer–Heyrovsky mechanism. The present addition of dispersive forces displaces peak Pt performance to negative Δ*G*_ads_ values, but trends are kept, in that *bcc* TMs display excessively strong Δ*G*_ads_ values for HER performance and *hcp* TMs are divided into too strongly and too weakly bound H* species. In that sense, the experimentally observed better performance of Pt-group TMs is well-kept. Still, the volcano shape of the experimental logarithm of the exchanged current density with the Δ*G*_ads_ estimates is not enough to allow for an unequivocal, quantitative performance analysis, with regressions deviating from the purely theoretical assessment, and with mean absolute errors of up to 2 orders of magnitude distant from experiments, so that such an assessment is recommended to be carried out in a qualitative fashion, to discard clear materials far from the Pt-group values.

## Supporting Information Available

(Tables S1–S3) Adsorption and absorption energies for all studied locations and surfaces on *bcc*, *fcc*, and *hcp* structures, respectively; (Figure S1) adsorption energy *vs* surface energy; (Figure S2) adsorption energy *vs* work function; (Figure S3) adsorption energy *vs* corrected *d*-band center; (Figure S4) adsorption energy *vs* highest Hilbert transform *d*-band peak; (Figure S5) volcano plot of overpotencial *vs* computed H* Δ*G*_ads_ for each TM averaging the stability of Wulff surfaces (PDF)
