# Supplementary material for: Atomic Hydrogen Interaction with Transition Metal Surfaces: A High-Throughput Computational Study
Source: J Phys Chem C Nanomater Interfaces. 2024 Nov 16;128(47):20129–39. doi: 10.1021/acs.jpcc.4c06194 (PMC11613584; doi:10.1021/acs.jpcc.4c06194)

Atomic Hydrogen Interaction with Transition Metal Surfaces: A High-Throughput Computational Study

Miquel Allés, Ling Meng, Ismael Beltrán, Ferran Fernández, and Francesc Viñes*

Departament de Ciència de Materials i Química Física & Institut de Química Teòrica i Computacional (IQTCUB), Universitat de Barcelona, c/ Martí i Franquès 1-11, 08028, Barcelona, Spain

* Corresponding author: francesc.vines@ub.edu

Table S1. Adsorption, E_{ads}, and absorption, E_{abs}, energies, on the studied *bcc* structure TMs surfaces. All values are given in eV.

TM	(001)	E_{abs}	E_{ads}	(011)	E_{abs}	E_{ads}	(111)	E_{abs}	E_{ads}
V	T	0.12	-0.02	T	-0.18	0.13	T	0.96	0.10
	B	-0.33	-0.65	B	-0.22	-0.82	B	-0.82	-0.74
	H	-0.74	-0.74	H	-0.99	-1.09	H_F	-0.33	-0.33
							H_H	-0.74	-0.56
Nb	T	0.32	-0.03	T	-0.10	0.28	T	1.25	0.12
	B	-0.27	-0.70	B	-0.12	-0.69	B	-0.62	-0.62
	H	-0.45	-0.45	H	-0.03	-0.95	H_F	0.00	0.00
							H_H	1.22	-0.16
Ta	T	0.43	-0.18	T	-0.06	0.10	T	1.18	-0.18
	B	-0.18	-0.75	B	-0.09	-0.77	B	-0.70	-0.73
	H	-0.44	-0.29	H	0.05	-1.01	H_F	0.03	0.03
							H_H	0.82	-0.35
Cr	T	0.43	-0.38	T	1.06	-0.03	T	1.05	-0.01
	B	-0.64	-1.74	B	0.80	-0.65	B	-0.77	-0.75
	H	-0.88	-0.88	H	0.66	-0.81	H_F	-0.31	-0.30
							H_H	0.11	-0.65
Mo	T	0.60	-0.07	T	0.93	-0.09	T	1.29	-0.08
	B	-0.23	-1.18	B	0.65	-0.70	B	-0.63	-0.63
	H	-0.60	-0.60	H	-0.58	-0.76	H_F	0.01	0.01
							H_H	-0.42	-0.42
W	T	0.84	-0.45	T	1.24	-0.18	T	1.84	-0.30
	B	-0.39	-1.86	B	0.94	-0.74	B	-0.59	-0.78
	H	-0.61	-0.61	H	-0.53	-0.76	H_F	0.16	0.16
							H_H	0.87	-0.42
Fe	T	0.17	0.12	T	0.47	-0.18	T	1.40	1.88
	B	-0.26	-0.48	B	0.34	-0.81	B	-0.48	-0.48
	H	-0.49	-0.96	H	-0.77	-0.76	H_F	1.94	1.94
							H_H	0.62	0.62

Table S2. Adsorption, E_{ads}, and absorption, E_{abs}, energies, on the studied *fcc* structure TMs surfaces. All values are given in eV.

**TM**	**(001)**	E_{abs}	E_{ads}	**(011)**	E_{abs}	E_{ads}	**(111)**	E_{abs}	E_{ads}
Ir	T	0.99	-0.64	T	1.64	-0.61	T	1.06	-0.51
	B	1.11	-0.74	H	0.00	0.00	B	-0.52	-0.47
	H	-0.42	-0.42	B_L	-0.22	-0.22	H_H	-0.52	-0.52
				B_S	0.93	-0.65	H_F	-0.50	-0.50
Pt	T	0.40	-0.53	T	0.52	-0.68	T	0.24	-0.59
	B	0.34	-0.75	H	-0.11	-0.11	B	-0.61	-0.60
	H	-0.38	-0.38	B_L	-0.34	-0.34	H_H	-0.61	-0.60
				B_S	-0.70	-0.70	H_F	0.19	-0.56
Pd	T	-0.19	-0.13	T	0.05	-0.13	T	-0.19	-0.13
	B	-0.18	-0.57	H	-0.27	-0.27	B	-0.25	-0.64
	H	-0.56	-0.56	B_L	-0.49	-0.49	H_H	-0.25	-0.64
				B_S	-0.23	-0.53	H_F	-0.22	-0.59
Rh	T	0.25	-0.28	T	0.87	-0.21	T	0.44	-0.21
	B	0.44	-0.58	H	-0.22	-0.22	B	0.15	-0.57
	H	-0.55	-0.55	B_L	-0.34	-0.34	H_H	0.15	-0.57
				B_S	0.27	-0.48	H_F	-0.55	-0.55
Ni	T	0.01	-0.09	T	0.59	0.00	T	0.22	-0.08
	B	0.22	-0.52	H	-0.34	-0.34	B	-0.02	-0.50
	H	-0.61	-0.61	B_L	-0.48	-0.48	H_H	-0.02	-0.64
				B_S	0.06	-0.46	H_F	0.15	-0.64
Cu	T	0.49	0.28	T	0.91	0.29	T	0.60	0.33
	B	0.59	-0.15	H	-0.01	-0.01	B	0.36	-0.28
	H	-0.23	-0.22	B_L	-0.16	-0.16	H_H	0.36	-0.28
				B_S	0.35	-0.26	H_F	-0.28	-0.28
Ag	T	0.71	0.62	T	1.11	0.65	T	0.91	0.64
	B	0.82	0.25	H	0.50	0.49	B	0.18	0.19
	H	0.25	0.25	B_L	0.29	0.29	H_H	0.18	0.19
				B_S	0.20	0.19	H_F	0.19	0.19
Au	T	0.99	0.25	T	1.05	0.25	T	0.73	0.29
	B	0.02	0.02	H	0.62	0.62	B	0.13	0.10
	H	0.33	0.33	B_L	0.27	0.27	H_H	0.09	0.10
				B_S	-0.01	-0.01	H_F	0.13	0.13

Table S3. Adsorption, E_{ads}, and absorption, E_{abs}, energies, on the studied *hcp* structure TMs surfaces. All values are given in eV.

**TM**	**(0001)**	$\boldsymbol{E_{abs}}$	$\boldsymbol{E_{ads}}$	**(10$\bar{1}$0)**	$\boldsymbol{E_{abs}}$	$\boldsymbol{E_{ads}}$	**(11$\bar{2}$0)**	$\boldsymbol{E_{abs}}$	$\boldsymbol{E_{ads}}$
Sc	T	-1.12	-1.07	T	-0.20	0.25	T	-0.82	-1.01
	B	-0.87	-1.14	H	-0.91	-0.19	H	-1.01	-1.01
	H	-0.91	-1.07	B_L	-0.97	-0.97	B_L	-1.11	-1.11
	H_E	-0.87	-1.14	B_S	-0.73	-1.03	B_S	-0.84	-0.71
Y	T	-1.17	-0.97	T	-0.32	0.25	T	-0.83	-0.92
	B	-0.79	-1.06	H	-0.91	-0.04	H	-0.92	-0.92
	H	-0.95	-0.97	B_L	-0.87	-0.87	B_L	-1.03	-1.03
	H_E	-0.78	-1.06	B_S	-0.77	-0.99	B_S	-0.79	-0.68
Ti	T	-0.42	-1.21	T	0.01	-0.05	T	-0.28	-0.80
	B	-0.56	-1.21	H	-0.46	-0.34	H	-0.80	-0.80
	H	-1.21	-1.20	B_L	-0.92	-0.91	B_L	-0.84	-0.43
	H_E	-0.56	-1.16	B_S	-0.35	-0.90	B_S	-0.58	-0.58
Zr	T	-0.51	-1.06	T	-0.20	-0.03	T	-0.33	-0.06
	B	-0.48	-1.11	H	-0.44	-0.10	H	-0.72	-0.83
	H	-0.57	-1.11	B_L	-0.79	-0.79	B_L	-0.80	-0.41
	H_E	-0.48	-1.06	B_S	-0.32	-0.83	B_S	-0.53	-0.66
Hf	T	-0.36	-1.13	T	-0.11	-0.12	T	-0.07	-0.18
	B	-0.42	-1.14	H	-0.36	-0.06	H	-0.65	-0.65
	H	-0.57	-1.13	B_L	-0.80	-0.80	B_L	-0.71	-0.32
	H_E	-0.42	-1.14	B_S	-0.24	-0.96	B_S	-0.32	-0.71
Tc	T	0.70	-0.76	T	0.84	0.92	T	0.66	-0.37
	B	0.09	-0.82	H	0.52	1.44	H	-0.37	-0.37
	H	-0.76	-0.76	B_L	0.54	-0.62	B_L	-0.35	-0.35
	H_E	0.09	-0.82	B_S	0.91	-0.80	B_S	0.30	-0.52
Re	T	0.87	-0.91	T	0.95	-0.31	T	1.15	-0.35
	B	0.19	-0.95	H	0.96	-0.13	H	-0.21	-0.21
	H	-0.90	-0.91	B_L	0.57	-0.55	B_L	-0.20	-0.20
	H_E	0.19	-0.95	B_S	0.94	-0.91	B_S	0.79	-0.72
Ru	T	1.01	-0.65	T	0.83	-0.28	T	0.55	-0.43
	B	0.29	-0.65	H	0.85	-0.67	H	-0.43	-0.43
	H	-0.58	-0.58	B_L	0.58	-0.61	B_L	-0.49	-0.49
	H_E	0.29	-0.65	B_S	0.57	-0.66	B_S	0.13	-0.59
Os	T	1.56	-0.44	T	1.22	-0.61	T	0.30	-0.55
	B	-0.61	-0.61	H	1.36	-0.21	H	-0.47	-0.47
	H	-0.55	-0.55	B_L	0.97	-0.33	B_L	-0.58	-0.58
	H_E	-0.61	-0.61	B_S	1.16	-0.79	B_S	0.74	-0.74
Co	T	0.45	-0.02	T	0.71	0.07	T	0.46	-0.40
	B	0.06	-0.64	H	0.49	-0.15	H	-0.40	-0.40
	H	-0.61	-0.61	B_L	0.53	-0.52	B_L	-0.39	0.08
	H_E	0.06	-0.64	B_S	0.49	-0.60	B_S	0.23	-0.46
Zn	T	0.93	0.50	T	0.77	0.56	T	0.67	0.20

	B	0.89	0.43	H	0.77	1.70	H	0.11	0.21
	H	0.89	0.58	B_L	0.90	0.49	B_L	0.28	0.22
	H_E	1.04	0.53	B_S	0.90	0.41	B_S	0.38	0.31
Cd	T	0.72	0.63	T	0.89	0.82	T	0.57	0.26
	B	0.68	0.53	H	0.88	0.83	H	0.52	1.55
	H	0.69	0.59	B_L	0.89	0.81	B_L	0.67	1.64
	H_E	0.74	0.55	B_S	0.90	0.75	B_S	0.71	0.24

Figure S1. Adsorption energy, E_{ads}, *vs.* surface energy, γ. The *bcc*, *fcc*, and *hcp* crystallographic structures are represented in red, blue, and green, respectively. The overall linear regression is shown, along with the regression coefficient, *R*.

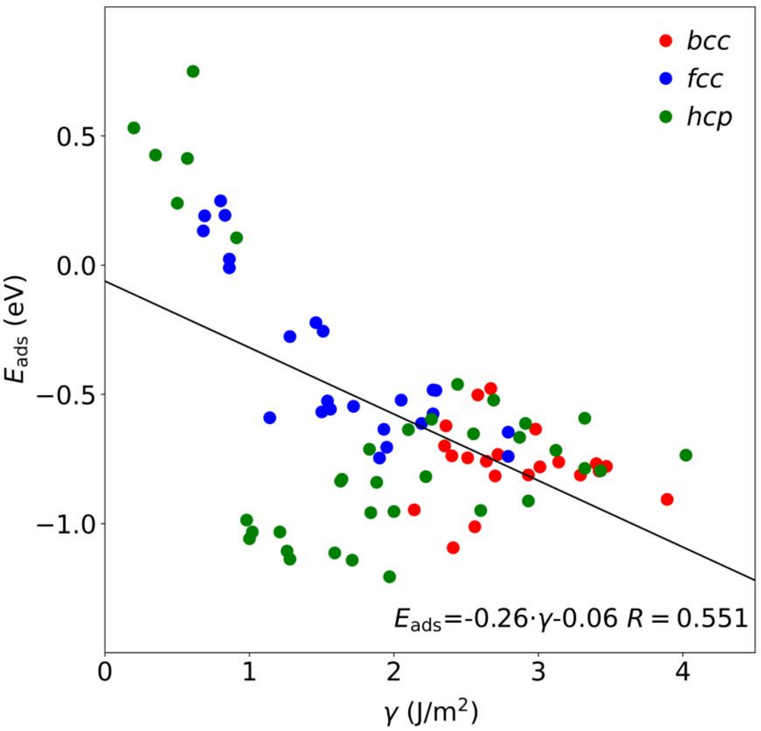

Figure S2. Adsorption energy, E_{ads}, *vs.* work function, ϕ. The *bcc*, *fcc*, and *hcp* crystallographic structures are represented in red, blue, and green, respectively. The overall linear regression is shown, along with the regression coefficient, *R*.

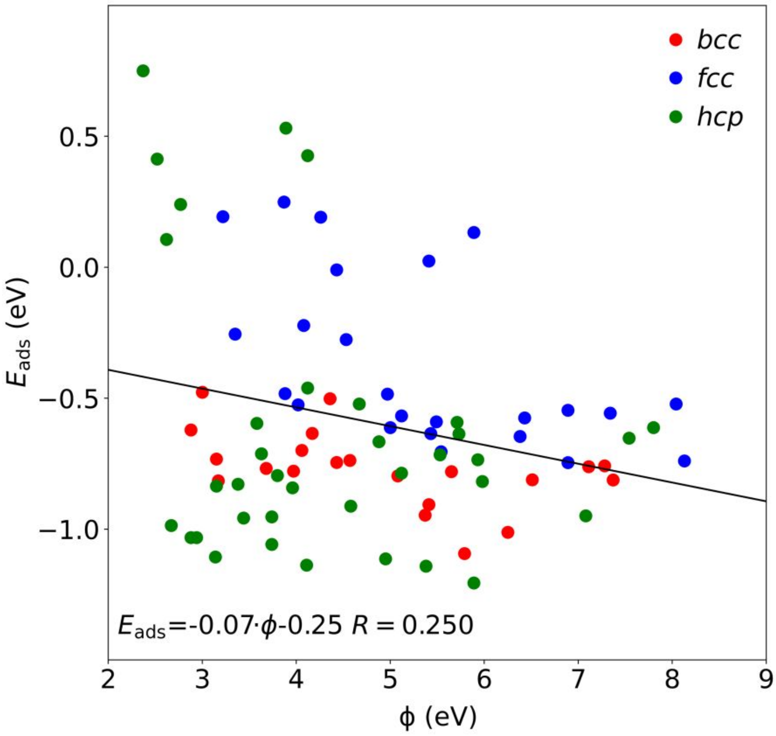

Figure S3. Adsorption energy, E_{ads}, *vs*. corrected *d*-band center, ε_d^W. The *bcc*, *fcc* and *hcp* crystallographic structures are represented in red, blue, and green, respectively. The overall linear regression is shown, along with the regression coefficient, *R*.

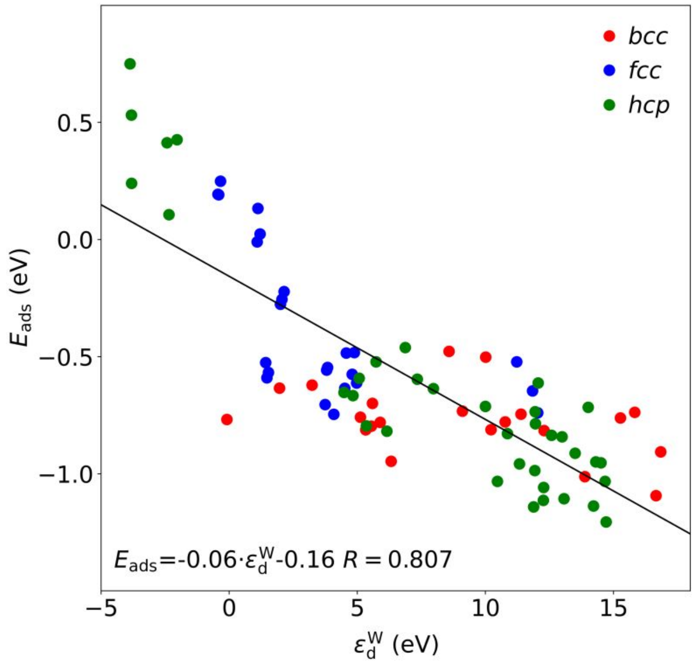

Figure S4. Adsorption energy, E_{ads}, *vs.* highest Hilbert transform *d*-band peak, ε_u. The *bcc*, *fcc* and *hcp* crystallographic structures are represented in red, blue, and green, respectively. The overall linear regression is shown, along with the regression coefficient, *R*.

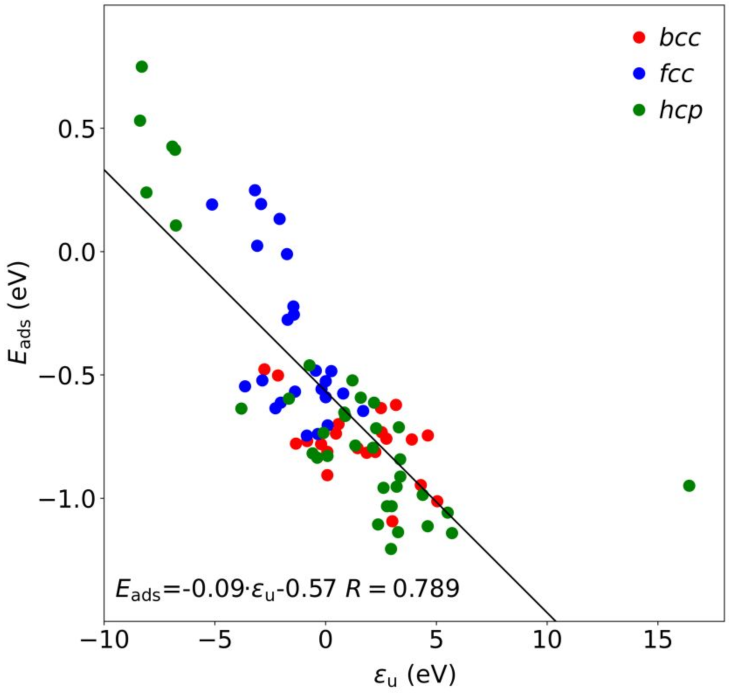

Figure S5. Volcano plot of overpotential, -η, *vs.* computed H* ΔG_{ads} for each TM averaging the stability of Wulff surfaces. The *bcc, fcc,* and *hcp* crystallographic structures are represented in red, blue, and green, respectively.

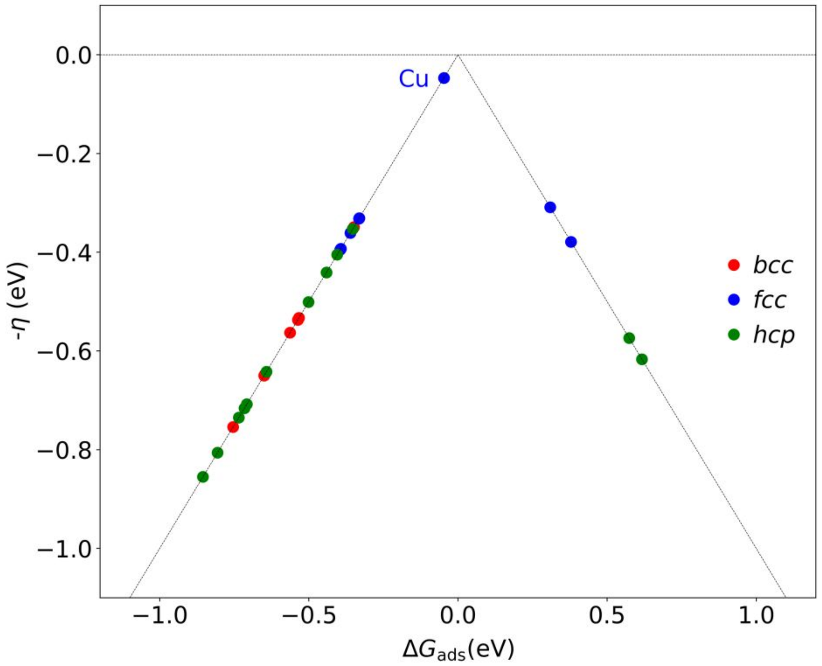

Supplement: Supplementary file 1 — jp4c06194_si_001.pdf [file jp4c06194_si_001.pdf]
